# Effect of apexification on occlusal resistance of immature teeth

**DOI:** 10.1186/s12903-020-01317-x

**Published:** 2020-11-12

**Authors:** WooCheol Lee, Yeon-Jee Yoo

**Affiliations:** 1 Department of Conservative Dentistry, Dental Research Institute, Seoul National University School of Dentistry, Seoul, Republic of Korea; 2 Department of Comprehensive Treatment Center, Seoul National University Dental Hospital, Daehakro 101, Jongno-Gu, Seoul, 03080 Republic of Korea

**Keywords:** Apexification, Immature tooth, Occlusal resistance, Strain gauge

## Abstract

**Background:**

Strain distribution was investigated to assess the occlusal resistance alterations in immature teeth under different occlusal force.

**Methods:**

In vitro apexification models of teeth with a funnel-shaped immature apex were obturated with mineral trioxide aggregate (MTA; ProRoot MTA) using different combinations of core materials (10/group): group 1, full-length orthograde obturation of MTA; group 2, a 5-mm MTA apical plug with a composite core; group 3, a 5-mm MTA apical plug and back-filling with warm gutta-percha. Teeth with calcium hydroxide (CH)-medicated canals and untreated teeth with normal apices were tested as controls. The teeth were arranged between two adjacent normal-apex teeth, embedded in a resin mold with a simulated periodontal ligament space. Strain data were recorded from the 3-unit teeth assembly under static compressive occlusal forces (50, 100, 200, and 300 N). Measurements were repeated 20 times for each condition, and the data were statistically analyzed.

**Results:**

The immature teeth showed altered occlusal force resistance, placing increased strain on adjacent teeth. Teeth with CH-medicated canals showed significantly inferior occlusal resistance under all tested forces (*P* < 0.05). Application of an MTA plug with deep composite resin core resulted in significantly better stress-bearing capacity especially under forces of 50 and 300 N (*P* < 0.05).

**Conclusions:**

The pattern of occlusal force distribution in immature teeth differed according to the canal obturation materials used for apexification. Immature teeth with an MTA apical plug showed more favorable occlusal force resistance than those with CH-medicated canals.

## Background

Regenerative endodontic procedures enable continued root growth, in both length and width, in immature teeth with pulp necrosis and apical periodontitis. However, it is not always possible to achieve successful root maturation. In particular, immature teeth with completely destroyed apical papillae or previous unsuccessful endodontic interventions fail to complete further root formation. An alternative treatment strategy in such cases is to induce apical barrier formation by apexification.

Concerns exist regarding the use of calcium hydroxide (CH) or mineral trioxide aggregate (MTA) as an intracanal medicament in apexification treatment, since these patients are usually relatively young. The conventional approach induces the formation of an apical barrier through long-term CH treatment [1, 2]. This treatment strategy involves multiple visits for medication changes and root canal irrigation over a prolonged period, which may lead to the loss of temporary dressings and re-infection. During the follow-up period, the high pH of the materials may weaken the dentin [3], making the tooth prone to failure [3–6]. Furthermore, cervical root fracture is a frequently-observed adverse event during apexification treatment and follow-up [5–7]. For this reason, several researchers have investigated possible methods of strengthening the tooth sufficiently to withstand the treatment period while maintaining proper function. In this regard, the use of MTA was advocated because of its proven favorable biological and sealing effects [8]. A general consensus exists regarding the use of MTA in regenerative endodontic procedures, especially for single-visit apexification, in which nonsurgical compaction of MTA into the apical end of the root canal can create an artificial apical stop and enable immediate obturation of the root canal [9].

Root reinforcement is a priority in the treatment of immature permanent teeth. Each occluding tooth and its root transmit a different amount of stress to the supporting architecture depending on its occlusal contact area, crown and root, location in dental arch, and occlusal scheme. When a tooth is replaced by a substitute, such as an open-apex tooth, its adjacent teeth will experience a different distribution of occlusal forces during mastication. However, to our knowledge, no previous study has investigated occlusal force resistance in teeth with immature root architecture. Therefore, the present study was conducted to investigate the effects of the apexification method on strain distribution in immature teeth under different occlusal forces.

## Methods

### In vitro apexification model

The sample size was calculated with G*Power 3.1.9 (Universität Kiel, Kiel, Germany) to detect significant differences (α = 0.05, 85% power). The estimated sample size was 10 teeth in each group. A total of 50 single-rooted extracted human lower premolars with similar crown and root dimensions were used in this study. Teeth with an intact crown and root that had not undergone any treatments were included. Teeth with cervical abrasions were excluded to minimize possible micro-flexion under loading. An in vitro apexification model was prepared according to a previous study [10]. The mean crown dimensions of the collected teeth were 8.2 ± 0.7 mm in the maximum mesio-distal dimension and 9.5 ± 0.8 mm in crown height measured from the cusp tip to the cementoenamel junction (CEJ) at the buccal aspect. The crown dimensions were standardized to a maximum mesio-distal dimension of 7.0 mm and a crown height of 8.0 mm from the CEJ. To standardize root length, we cut approximately 3 mm of the apical root tip, resulting in an 8.0-mm root length below the CEJ measured at the buccal surface.

The teeth were accessed with a round carbide bur #4 (Komet, Rock Hill, SC), and their root canals were enlarged with a Gates Glidden bur #5 (Mani, Utsunomiya, Japan) to the working length. A simulated divergent open apex was generated by retrograde preparation with a tapered diamond bur (CR-11F, Mani, Utsunomiya, Japan). The root canals were irrigated with a 5.25% sodium hypochlorite (NaOCl) solution between instrumentation, and immersed in a 17% ethylenediaminetetraacetic acid solution (pH 7.2) for 1 min before a final flush with 5.25% NaOCl solution. All irrigants were activated using intracanal ultrasonic devices (P5 Newtron XS; Satelec Acteon group, Mérignac, France). Finally, the canals were copiously rinsed with sterile distilled saline and dried with sterile paper points.

The teeth were randomly assigned to the following experimental groups and obturated accordingly: group 1, full-length orthograde obturation of MTA (ProRoot MTA; Dentsply Tulsa Dental, Tulsa, OK); group 2, a 5-mm MTA apical plug with a composite core; and group 3, a 5-mm MTA apical plug and back-filling with warm gutta-percha (GP). MTA obturation was performed using the obturation technique suggested by Bogen and Kuttler [11]. ProRoot MTA was mixed with distilled water according to the manufacturer’s instructions and placed incrementally with a carrier gun. An SS K-file with a #100 size was used to compact the apical 3–4 mm, then progressively larger K-files were used for further compaction. The final coronal portion was tamped by using stainless steel hand pluggers to complete the root canal obturation. Proper placement and thickness were verified by radiographic examinations. Following obturation, the teeth were stored at 37 °C under 100% humidity for a week to allow complete setting of the filling materials. The access cavities were sealed using resin composite (Z250; 3 M ESPE, St Paul, MN) and bonding agent (Scotchbond multipurpose; 3 M ESPE). Teeth with CH-medicated canals (CleaniCal, Maruchi, Wonju, Korea) and untreated teeth with normal apices were tested as controls (n = 10 each). Teeth were stored at 37 °C with 100% humidity for 3 weeks until further analysis.

### Configuration of the instrument for strain measurement under occlusal forces

**Fig. 1:**
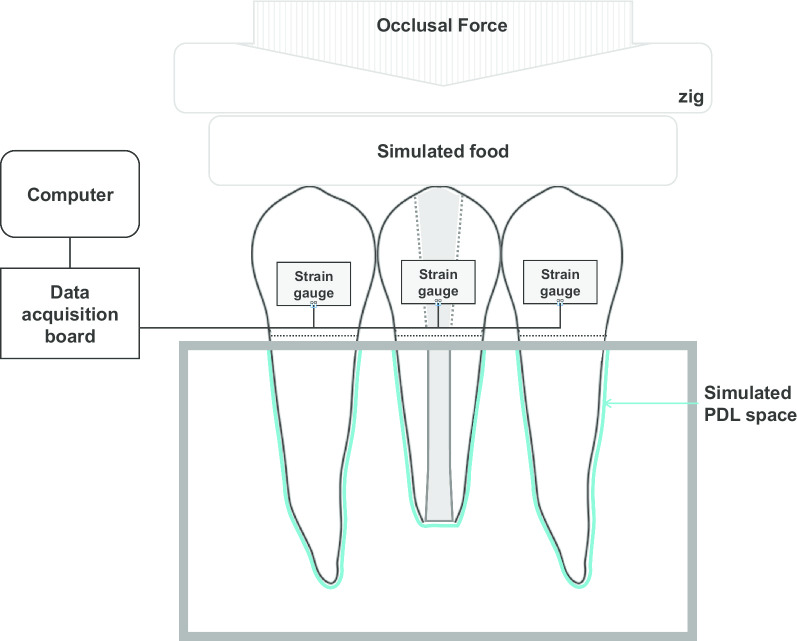
Schematic diagram of the 3-unit teeth assembly for strain analyses. PDL, periodontal ligament

The instrument for measuring strain development under occlusal forces consisted of a 3-unit teeth assembly constructed with extracted human teeth, strain gauges, and a data acquisition board (Fig. 1). Two intact teeth with normal apices (adjacent teeth) and the treated tooth (experimental tooth) were arranged in a straight line in a single occlusal plane and embedded in a mold. The roots were double-covered with 0.13-mm paraffin film (Parafilm M; Sigma-Aldrich, St. Louis, MO) to simulate a periodontal ligament (PDL) space of approximately 0.25-mm thickness. Strain gauges (KFG-2-350-C1-11, Kyowa Electronic Instruments, Tokyo, Japan) were attached to the buccal surfaces of the teeth at 4 mm below the buccal cusp tip. Simulated food fabricated with a polyvinylsiloxane impression material (Exafine putty type, GC Corp., Tokyo, Japan) was placed between the occlusal plane of the teeth and the test zig of the universal testing machine (Instron 8871, Instron Co., Norwood, MA) [12].

### Measurement of strain under occlusal force

The 3-unit teeth assembly model was calibrated to ensure that it was free of residual strain under default conditions (0 N). Data from the untreated teeth with normal apices were used to confirm the even distribution of occlusal stress under tested forces. Compressive occlusal forces of 50, 100, 200, and 300 N were applied with a universal testing machine, and strain values were simultaneously measured and recorded from each tooth. Strain measurements were repeated 20 times for each condition. The simultaneously recorded electronic measurements from each strain gauge were converted into occlusal force (N) measurements for each tooth. The occlusal resistance of each tooth was calculated as the percentage (%) of the occlusal force loaded in the treated tooth to the average occlusal force in the 3-teeth assembly, calculated from the summed values for all three strain gauges.

### Statistical analysis

All analyses were performed using SPSS version 19 (IBM Corp., Armonk, NY, USA). Occlusal resistance data were pooled according to the apexification method and tested occlusal forces. All data were analyzed using two-way analysis of variance (ANOVA) with the type of apexification method and occlusal force as the main factors, followed by Tukey’s post-hoc comparisons. For each tested occlusal force, data were separately analyzed with one-way ANOVA and Tukey’s post-hoc comparisons. The significance level was set at α = 0.05.

## Results

None of the tested teeth fractured during the experiment. Each summed occlusal force calculated from the three strain gauges was smaller than the applied occlusal forces, indicating stress absorption from tooth-supporting structures (e.g., simulated food, PDL, and mold) with a quadratic trendline (*R*^2^ = 0.95) in association with increasing occlusal force (Table 1).

**Table 1:** Occlusal force loaded on the teeth and stress absorbed by other components of the 3-unit teeth assembly under different occlusal forces

	Applied occlusal force (N)			
	50	100	200	300
Total occlusal force loaded on teeth (N)	44.98 ± 3.58	81.20 ± 5.83	153.34 ± 5.40	185.85 ± 17.01
Percentage (%) of stress absorption from simulated food and tooth-supporting structures	10.04 ± 7.16	18.79 ± 4.32	23.32 ± 5.70	38.04 ± 5.67

**Fig. 2:**
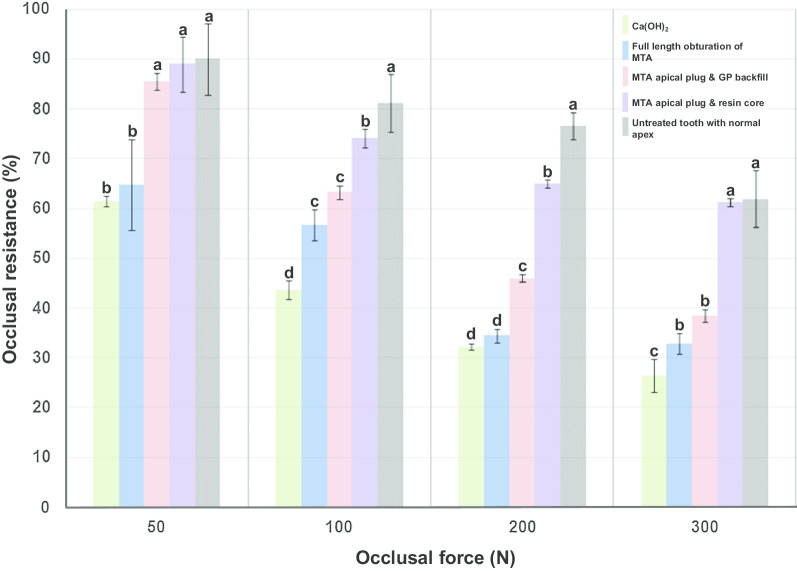
Occlusal resistance (%) of immature teeth treated with different apexification methods. MTA, mineral trioxide aggregate; GP, gutta-percha

All teeth in the simulated in vitro apexification model showed significantly lower occlusal resistance than the adjacent teeth with normal apex regardless of applied occlusal forces (*P* < 0.01). Teeth with CH-medicated canals showed least occlusal resistance under all tested forces than teeth that underwent other treatments (*P* < 0.05). Two-way ANOVA suggested that both factors (apexification method and applied occlusal forces) and their interaction were significant (*P* = 0.000). Teeth with 5-mm MTA plugs with deep composite cores (group 2) showed significantly better stress bearing capacity under all tested forces than the other apexification models (*P* < 0.05). Under 50- and 300-N occlusal forces, the teeth in group 2 showed similar occlusal resistance to adjacent teeth with normal apices (*P* > 0.05). The full-length MTA-filled roots showed significantly lower occlusal resistance under forces of 50- and 200-N than the other two groups that had been backfilled with either GP or composite resin. The occlusal resistances (%) of immature teeth treated with different apexification methods under each different occlusal force are presented in Fig. 2.

## Discussion

To date, no study has comprehensively determined changes in occlusal resistance in immature teeth, although some studies have estimated the strength of immature teeth, typically using ultimate fracture strength [13–16]. Load-to-failure testing is a simple way to estimate the ultimate strength of a specimen, but the test setting (e.g., cross-head speed or load angle) varies widely, and testing assemblies exclude the physiological environment (such as PDL space). Finite element analysis is another approach to stress analysis that uses 2- or 3-dimensional computer simulated models. This approach is useful for understanding stress distribution within a simulated graphic model, but interpretation of the results can be difficult because this method assumes that the tooth structure is an isotropic and homogeneous material. The results differ according to the type of pre-set model (e.g., linear or nonlinear, plastic or elastic). These methods cannot represent occlusal force distribution and resistance in clinical settings; hence, they have limited relevance in predicting the clinical performance of immature teeth.

In this study, we used human teeth with strain gauges attached to measure the actual stress that they experienced under occlusal forces on an immature tooth during and after apexification. The tested tooth was aligned in a 3-unit teeth assembly simulating adjacent teeth and the PDL space as a stress breaker. It has been reported that the average chewing force varies from 11 to 150 N, whereas the force peaks are 200 N in the anterior area, 350 N in the posterior area, and 1000 N in patients with bruxism [17]. In another study, the maximum bite forces exhibited by adults in the molar area ranged from 250 to 400 N, and forces in the anterior area ranged from 140 to 170 N [18]. Therefore, we preset the occlusal forces as 50 to 300 N.

All simulated immature teeth showed significantly less strain under all tested occlusal forces, and the CH-medicated canals showed the lowest values among the tested teeth. The inferior occlusal resistance of CH-filled teeth may be due to the absence of an apical plug to resist compressive forces. CH in a non-set, aqueous suspension is recommended as the material of choice for apexification [1, 2]. Despite the unpredictability of apical closure, intracanal CH medication can prevent the ingress of granulation tissue into the root canal and inhibit periapical osteoclastic activity [1, 2]. Occlusal force resistance might have different patterns if an apical calcified barrier is formed, but it was not possible to simulate an irregular porous apical barrier in the in vitro setup.

MTA-filled immature teeth with full-length roots also transmitted occlusal stress to the adjacent teeth. Owing to the slow hydration rate of dicalcium silicate, which is one of the main components of MTA, occlusal force measurements were performed 3 weeks after specimen preparation to maximize the compressive strength of the material [19]. MTA is known to form a superficial apatite layer and induce intratubular mineralization that are supposed to enhance sealability of the material [20–25]. However, the firmly set material with its micromechanical retentive form in the root dentin was not directly connected to improved resistance to compressive occlusal forces. This might have been because the MTA surface acted as a stress endpoint, transmitting the strain to the adjacent canal wall. If the material surface ends at the cervical area where the enamel ends and the force accumulates, the tooth will not be able to resist the occlusal stress.

For this reason, GP and a composite restoration were placed as reinforcement materials at the cervical area of the immature teeth. The GP backfill added more interfaces inside the canal, and failed to reinforce the thin canal wall. However, a deep composite restoration extending from the MTA plug surface to the occlusal top provided improved occlusal force resistance, which was comparable to that of normal mature teeth. The results of CH-medicated roots could be interpreted as showing that the internal interface at the cervical area adversely affects the occlusal force resistance of immature teeth.

Considering the importance of the periodontium in supporting teeth during function, the depleted occlusal force resistance of immature teeth during a prolonged treatment period might result in stress shielding of the periapical area of the treated tooth, thereby potentially slowing the healing of periapical inflammation and remodeling process of the alveolar bone proper [26–28]. It was also reported that the reduction of occlusal force altered the development and maintenance of mechanoreceptors such as Ruffini endings in the PDL [29–31].

A limitation of the current experiment is that only compressive forces were simulated. When a tensile force is loaded, we speculate that the situation will be more complex. Considering the compressive yield strength of dental tissues, strain development under compressive occlusal forces would not be significant enough to cause immediate tooth failure [32]. However, when a lateral force is applied, higher tensile stress will be generated than when a compressive load is applied to the same area. Given the thin root wall of immature teeth, such tensile overloading may cause cracks or even root fracture. Therefore, proper adjustments of occlusal surfaces should be performed to prevent these events.

Within the limitations of this study, it was confirmed that the pattern of occlusal force distribution in immature tooth differed according to the canal obturation material used for apexification. Application of an MTA plug with a deep composite resin core could increase the occlusal force resistance of immature teeth during and/or after apexification treatment. Further in-depth studies are required to optimize apexification procedures in terms of clinical performance during the treatment period.

## Conclusions

Under the conditions of this in vitro study, it was concluded that:

1. Occlusal force distribution varied in immature teeth depending on the root canal obturation material used for apexification.
2. Application of an MTA plug with a deep composite resin core could increase the occlusal force resistance of immature teeth during and/or after apexification treatment.

## Data Availability

All data used and/or analyzed during this research are available from the corresponding author on reasonable request.

## Abbreviations

CEJ: Cemento-enamel junction
CH: Calcium hydroxide
GP: Gutta-percha
MTA: Mineral trioxide aggregate
PDL: Periodontal ligament
